# Genome sequencing and comparison of five *Tilletia* species to identify candidate genes for the detection of regulated species infecting wheat

**DOI:** 10.1186/s43008-019-0011-9

**Published:** 2019-07-24

**Authors:** Hai D. T. Nguyen, Tahera Sultana, Prasad Kesanakurti, Sarah Hambleton

**Affiliations:** 1 Biodiversity and Bioresources, Ottawa Research and Development Centre, Agriculture and Agri-Food Canada, 960 Carling Ave, Ottawa, Ontario K1A 0C6 Canada; 2 Present Address: Research Farm – Vineland, London Research and Development Centre, Agriculture and Agri-Food Canada, 4902 Victoria Avenue N., Vineland Station, Ontario L0R 2E0 Canada; 3 Present Address: NHP Research Alliance, College of Biological Sciences, University of Guelph, 50 Stone Road East, Guelph, Ontario N1G 2W1 Canada

**Keywords:** *Tilletia indica*, *Tilletia walkeri*, *Tilletia controversa*, *Tilletia caries*, *Tilletia laevis*, Comparative genomics, High throughput next generation sequencing, Phylogenomics

## Abstract

*Tilletia* species cause diseases on grass hosts with some causing bunt diseases on wheat (*Triticum*). Two of the four species infecting wheat have restricted distributions globally and are subject to quarantine regulations to prevent their spread to new areas. *Tilletia indica* causes Karnal bunt and is regulated by many countries while the non-regulated *T. walkeri* is morphologically similar and very closely related phylogenetically, but infects ryegrass (*Lolium*) and not wheat. *Tilletia controversa* causes dwarf bunt of wheat (DB) and is also regulated by some countries, while the closely related but non-regulated species, *T. caries* and *T. laevis*, both cause common bunt of wheat (CB). Historically, diagnostic methods have relied on cryptic morphology to differentiate these species in subsamples from grain shipments. Of the DNA-based methods published so far, most have focused on sequence variation among tested strains at a single gene locus. To facilitate the development of additional molecular assays for diagnostics, we generated whole genome data for multiple strains of the two regulated wheat pathogens and their closest relatives. Depending on the species, the genomes were assembled into 907 to 4633 scaffolds ranging from 24 Mb to 30 Mb with 7842 to 9952 gene models predicted. Phylogenomic analyses confirmed the placement of *Tilletia* in the *Exobasidiomycetes* and showed that *T. indica* and *T. walkeri* were in one clade whereas *T. controversa*, *T. caries* and *T. laevis* grouped in a separate clade. Single copy and species-specific genes were identified by orthologous group analysis. Unique species-specific genes were identified and evaluated as suitable markers to differentiate the quarantine and non-quarantine species. After further analyses and manual inspection, primers and probes for the optimum candidate genes were designed and tested in silico, for validation in future wet-lab studies.

**Electronic supplementary material:**

The online version of this article (10.1186/s43008-019-0011-9) contains supplementary material, which is available to authorized users.

## INTRODUCTION

There are approximately 140 known species in the fungal genus *Tilletia* (*Tilletiales*, *Ustilaginomycotina*), all causing diseases on grass hosts in the *Poaceae* family. Two of the four species infecting wheat (*Triticum*), *T. indica* and *T. controversa* (as *T. ‘contraversa’*), are subject to quarantine regulations in various countries while *T. caries* and *T. laevis* on wheat and other grasses are also of major concern for agricultural production (Carris et al. 2006). *Tilletia* species vary in their infection process but sporulation for most occurs in the plant ovary with host tissues in the kernel gradually replaced by masses of darkly pigmented teliospores, so-called “bunt balls”. Morphological identification relies on often subtle differences in teliospore colour, size and wall ornamentation, and the presence or absence of a pale sheath (Wilcoxson and Saari 1996). Some diagnostic procedures require spores to be first germinated in the lab which may take several weeks, with success dependent on appropriate growth conditions and spore viability.

Phylogenetic analyses of nuclear ribosomal (rDNA) large subunit sequences (LSU) demonstrated that the four pathogens on wheat and other grasses, and other species occurring on hosts also in the subfamily *Pooideae*, form a large and well-supported clade within a monophyletic genus (Castlebury et al. 2005). Furthermore, this large clade was subdivided, with *T. indica* and its closest relative *T. walkeri* on ryegrass (*Lolium*) in their own well-supported sub-clade with identical sequences. The other three wheat-infecting species were grouped with a dozen other species occurring on other hosts, also in a well-supported subclade but with few nucleotide differences amongst species.

*Tilletia indica* causes the disease of wheat commonly called Karnal bunt. Many countries, including Canada, have a zero tolerance for Karnal bunt spores in wheat seed importations. Symptoms are very difficult to detect in the field and may not be observed until harvest and, although the disease might cause only a small reduction in yield, it reduces grain quality making it unsatisfactory for human consumption (IPPC 2016). During the 1996 National Karnal Bunt Survey in the United States, spores morphologically like those of *T. indica* were discovered in washes derived from wheat seed samples from southeastern states and in forage-mix seed lots from Oregon but no infected wheat seeds were found (Bonde et al. 1997; Cunfer and >Castlebury 1999). Further investigations led to the description of *T. walkeri* (Castlebury and Carris 1999), a species with similar teliospore morphology but infecting annual ryegrass, which may occur as a weed in areas of wheat production and be present in grain shipments as a contaminant (Castlebury et al. 2005).

Distinguishing *T. indica* from *T. walkeri* (and other species) is essential for international trade activities but morphological identification requires examination of spores by trained mycologists and the two species are very closely related genetically, with ITS fungal barcode sequences differing at only two positions (Levy et al. 2001). The most recent internationally accepted diagnostic protocols for *T. indica* were adopted by the International Plant Protection Convention (IPPC) in 2014 and published in 2016 (IPPC 2016). Of the four molecular methods adopted in that standard, three depend on germination of teliospores for sufficient DNA concentrations (Pimentel et al. 1998; Levy et al. 2001; Frederick et al. 2000). The fourth is a multiplex real-time ITS PCR assay (Tan et al. 2009) for multiple *Tilletia* species but was designed with an initial PCR amplification step for DNA extracted directly from single crushed teliospores. More recently published LAMP (Loop Mediated Isothermal Amplification) assays target unique regions in the mitochondrial DNA (Gao et al. 2016; Tan et al. 2016).

*Tilletia controversa* causes dwarf bunt of wheat (DB) and has a restricted distribution while *T. caries* (syn. *T. tritici*) and *T. laevis* (syn. *T. foetida*) both cause common bunt of wheat (CB) and are widely distributed globally in wheat-growing regions. The three species are very closely related genetically and biologically, and cannot be accurately identified using ITS barcodes. While analyses based on ITS, EF1a and RPB2 showed variation among sequences for DB compared to CB, support was lacking and sampling was limited (Carris et al. 2007). Importantly, *T. controversa* can cause major yield losses and is an internationally quarantined pathogen to prevent its entry to new areas (OEPP/EPPO 2016) and CB, which has been successfully controlled with chemical seed treatments for many years, has made a resurgence under low-input and organic production (Borgen and Davanlou 2001; Matanguihan et al. 2011; Župunski et al. 2012).

The toolkit of DNA sequence based assays for the regulated species is as yet limited to one or a few options and gene regions, especially for DB (Liu et al. 2009; Zouhar et al. 2010; Gao et al. 2014) and CB (Josefsen and Christiansen 2002; Kochanová et al. 2004) and studies demonstrating their efficacy and specificity during regulatory inspection of field samples are lacking. Thus, despite the known limitations inherent in morphology-based identifications, federal diagnostic labs continue to rely on labour- and time-intensive seed-washing and microscopy techniques (IPPC 2016) to screen grain importations for teliospores of regulated *Tilletia* species, at least in Canada. Comparative genomics has the potential to identify multiple gene regions that can be targeted for assay development, potentially at hierarchical or tiered levels of phylogenetic resolution and redundancy, to ensure false negatives are avoided and to detect new or novel genotypes.

Although no genome assemblies were publicly available for the genus *Tilletia* when this study was initiated, there are now genomes for *T. indica* or *T. walkeri* (Tan et al. 2016; Sharma et al. 2016; Kumar et al. 2017; Kumar et al. 2018) and *T. horrida* (Wang et al. 2015), but no other species. We have sequenced, annotated and compared the whole genomes of three strains of *T. indica*, two strains of *T. walkeri*, two strains of *T. controversa*, one strain of *T. caries* and two strains of *T. laevis*. Our objective was to search for candidate genes that were shared amongst, between and within species, in addition to those previously targeted for diagnostics. Of particular interest to achieve in this study was to identify a set of genes unique to each species, as candidate loci for future real time PCR assay development, with the focus on robust differentiation of non-quarantine from quarantined species for regulatory purposes.

## MATERIALS AND METHODS

### Growth, DNA/RNA extraction and sequencing

Ten *Tilletia* strains representing five *Tilletia* species were selected for this study: *T. caries* DAOMC 238032, *T. controversa* DAOMC 236426 & DAOMC 238052, *T. indica* DAOMC 236414, DAOMC 236408 & DAOMC 236416, *T. laevis* DAOMC 238040 & ATCC 42080, and *T. walkeri* DAOMC 236422 & DAOMC 238049. The DAOMC strains were cultured as polysporidial isolates from surface-sterilized germinated teliospores by the Canadian Food Inspection Agency and most were included in a study by McDonald et al. (2000). They were later provided to Agriculture and Agri-Food Canada as pure cultures for research purposes and for long term preservation in the Canadian Collection of Fungal Cultures (DAOMC) in Ottawa, Canada. The ATCC culture was obtained from the American Type Culture Collection (Manassas, VA USA).

DNA was extracted from mycelia grown on solid potato dextrose agar (PDA; Difco, Becton Dickinson, NJ, USA) at room temperature in the dark and using one of the following kits with the manufacturer’s instructions: E.Z.N.A.® Fungal DNA Miniprep kit (VWR, Mississauga, ON, Canada), OmniPrep for Fungi kit (G-Biosciences, St. Louis, MO, USA), or Macherey-Nagel Nucleospin® 96 Plant or Macherey-Nagel NucleoMag® 96 Trace kit (Macherey Nagel GmbH & Co. KG, Düren, Germany). One representative of each species, except for *T. laevis*, was also grown for RNA extraction: *T. caries* DAOMC 238032, *T. controversa* DAOMC 236426, *T. indica* DAOMC 236416 and *T. walkeri* DAOMC 236422. They were grown on solid PDA plates at room temperature in the dark and in three liquid media preparations under natural light at 20 °C and at 120 rpm on a rotary shaker: T19 medium (customized for *Tilletia*; Trione 1964), potato-sucrose agar (from diced potatoes; Wilcoxson and Saari 1996), and 2% Malt agar with trace elements (Samson et al. 2010). RNA was extracted using the Nucleospin® RNA L (Midi) or Nucleospin® RNA II (Mini) extraction kits (Macherey Nagel GmbH & Co. KG, Düren, Germany) following the manufacturer’s instructions. DNA quality and quantity were checked using a Qubit® 2.0 Fluorometer (Invitrogen, ThermoFisher Scientific, Waltham, MA USA) on agarose gels and by ITS DNA barcode sequencing (data not shown). RNA quality and quantity were checked with Qubit and a Bioanalyzer (Agilent, Santa Clara, CA USA). Paired end sequencing (2 × 101 bp) of genomic libraries and RNA libraries of *T. caries* DAOMC 238032, *T. controversa* DAOMC 236426, *T. indica* DAOMC 236416 and *T. walkeri* DAOMC 236422 was performed on an Illumina HiSeq 2500 at the National Research Council Canada in Saskatoon, Saskatchewan, Canada. Preliminary unpublished versions of these assemblies and annotations were made available on NCBI in 2016 but these data have been superseded by the versions published here. Paired end sequencing (2 × 300 bp) of genomic libraries of *T. controversa* DAOMC 236426 and DAOMC 238052, *T. laevis* DAOMC 238040 and ATCC 42080, *T. indica* DAOMC 236408 and DAOMC 236414, and *T. walkeri* DAOMC 238049 was performed on an Illumina MiSeq at the Molecular Technologies Laboratory at Agriculture and Agri-Food Canada. A total of 20 Illumina libraries were generated. PacBio RS II sequencing of *T. controversa* DAOMC 236426 was performed at Genome Quebec in Montreal using two SMRT cells. The libraries generated in this study are summarized in Additional file 1: Table S1.

### Genome assembly and annotation

Prior to genome assembly, quality of reads from genomic DNA was checked with FastQC v0.10.1 (http://www.bioinformatics.babraham.ac.uk/projects/fastqc/). Adaptor sequences and poor quality bases were trimmed with Trimmomatic v.0.36 (Bolger et al. 2014). De novo sequence assembly was performed using SPAdes 3.7.1 (Bankevich et al. 2012), where k-mer sizes were automatically chosen based on input read lengths, with error correction using BayesHammer (Nikolenko et al. 2013) and with mismatch correction enabled. If a strain was sequenced multiple times, all datasets for that strain were specified in one assembly. Scaffolds shorter than 1000 bp were discarded. GapFiller v. 1.10 (Boetzer and Pirovano 2012) was used to close gaps in the scaffolds using the trimmed and corrected paired-end reads from above. Assembly statistics were generated with QUAST v2.3 (Gurevich et al. 2013). The corrected reads were mapped back onto the scaffolds using Bowtie2 v2.0.0 (Langmead and Salzberg 2012) and approximate nucleotide wise coverage was determined with Qualimap v2.2.1 (García-Alcalde et al. 2012).

To perform genome annotation, the quality of the sequenced reads from RNA was checked with FastQC v0.10.1, adaptor sequences and poor quality bases were trimmed with Trimmomatic v.0.36 as described above. These trimmed RNA reads were then mapped to the genome of the respective *Tilletia* species with TopHat v2.0.5 (Kim et al. 2013). RNA reads from *T. caries* DAOMC 238032 were mapped to the genomes of *T. laevis* because no RNA data were generated for that species. Following the mapping of RNA to the genome assemblies, gene prediction was performed by BRAKER v1.9 (Hoff et al. 2016) with the fungal option turned on and the alternative splicing determination option turned off. The BUSCO v 2.0 (Simão et al. 2015) program was used to assess genome assembly and annotation completeness with fungal profiles (http://busco.ezlab.org/v2/datasets/fungi_odb9.tar.gzk).

To determine potential function, the protein sequences from the UniProt/Swiss-Prot manually curated protein data set were compared with the annotated proteins of each *Tilletia* genome by blastp v2.2.26+ (Camacho et al. 2009). The gene models with BLAST hits having e-value of less than 1.0E^− 100^ and mean similarity hit of ≥90% were assumed to be orthologs and they were given names following recommended conventions (http://www.uniprot.org/docs/proknameprot). Annotations were validated using Genome Annotation Generator (Hall et al. 2014) and tbl2asn (http://www.ncbi.nlm.nih.gov/genbank/tbl2asn2/) was used to produce GenBank files for subsequent analyses and SQN files for genome submission to NCBI. Genes predicted from each genome were compared in pairs using MUMmer v. 3.23 (Kurtz et al. 2004). Using the predicted genes nucleotide sequences as input, the Average Nucleotide Identity (ANI) was calculated using FastANI (Jain et al. 2018).

Statistics for all ten *Tilletia* genomes are summarized in Table 1. Accession numbers for raw NGS data and for genome assemblies/annotations are provided in Additional file 1: Table S1.

**Table 1:** Genome statistics of *Tilletia* strains compared in this study

	*T. indica*	*T. indica*	*T. indica*	*T. walkeri*	*T. walkeri*	*T. controversa*	*T. controversa*	*T. caries*	*T. laevis*	*T. laevis*
DAOMC culture number	DAOMC 236408	DAOMC 236414	DAOMC 236416	DAOMC 236422	DAOMC 238049	DAOMC 236426	DAOMC 238052	DAOMC 238032	DAOMC 238040	ATCC 42080
Isolation source	*Triticum*	*Triticum*	*Triticum*	*Lolium*	*Lolium*	*Triticum*	*Triticum*	*Triticum*	*Triticum*	*Triticum*
Isolation location	India	Pakistan	Pakistan	Oregon, USA	Oregon, USA	Ontario, Canada	Ontario, Canada	Idaho, USA	Australia	Washington, USA
Sequencing method	Illumina MiSeq	Illumina MiSeq	Illumina HiSeq	Illumina HiSeq	Illumina MiSeq	Illumina HiSeq, MiSeq, PacBio	Illumina MiSeq	Illumina HiSeq	Illumina MiSeq	Illumina MiSeq
Number of scaffolds	2555	4624	3113	1387	907	3741	4243	4633	4501	3961
Largest scaffold size (bp)	158,844	75,904	161,394	260,724	330,910	114,021	81,512	72,392	101,287	101,260
N50 in scaffolds	30,694	11,729	23,649	45,552	79,486	14,874	12,786	11,482	11,569	13,920
Gaps per 100 kb	41.79	14.33	54.93	22.62	20.08	96.12	43.23	80.78	101.48	31.02
GC (%) in scaffolds	54.8	55.0	54.9	54.9	54.9	56.5	56.7	56.7	56.6	56.6
Total length (Mb) in scaffolds	29.7	29.0	29.0	24.0	24.3	29.9	28.6	28.1	28.3	28.8
Coverage^a^	51x	45x	38x	75x	46x	67x	45x	52x	67x	35x
Number of gene models	9410	9677	9664	7881	7842	9887	9649	9952	9651	9799
BUSCO (complete single copy)^b^	98%	93%	96%	97%	98%	95%	96%	92%	93%	95%

^a^Determined by mapping reads with Bowtie2 and analysis with Qualimap v2.1

^b^BUSCO analysis on scaffolds ≥1000 bp running on the fungal dataset

### Orthologous group analysis

For each *Tilletia* genome, genes (that include both exons & introns) and protein sequences were extracted as fasta files with an online tool (https://rocaplab.ocean.washington.edu/tools/genbank_to_fasta/) using GenBank files as input. Orthologous group analysis was performed with OrthoFinder v1.1.8 (Emms and Kelly 2015), on default settings, using protein sequences from each *Tilletia* genome as inputs. The mcl program (Enright et al. 2002) is part of the OrthoFinder pipeline and it is used to cluster similar proteins into groups called orthogroups that essentially represent putative gene families. A visual representation was made using InteractiVenn (Heberle et al. 2015) to illustrate the number of orthogroups shared between species and those unique to each species.

### Phylogenomics

To estimate relationships between the ten *Tilletia* genomes and to verify the species tree, orthogroups containing single copy genes shared between all *Tilletia* species were identified. Gene sequences at the nucleotide level were extracted as fasta files with the filterbyname.sh script from BBTools v35 (https://jgi.doe.gov/data-and-tools/bbtools/) where one fasta file was generated per orthogroup. Sequences were aligned with MUSCLE v3.8.31 (Edgar 2004) on default settings. The alignment statistics were measured with AMAS (Borowiec 2016). A maximum likelihood phylogenetic analysis was performed on each alignment with RAxML v8.2.9 (Stamatakis 2014) using the GTRGAMMA model with the fast bootstrap method (option -f a) and 100 bootstrap replicates. All generated trees were combined together and analysed in ASTRAL-II v4.10.10 (Mirarab and Warnow 2015) to construct a greedy consensus tree.

To estimate the phylogenetic position of *Tilletia* species in the *Ustilagomycotina*, we performed a phylogenomic analysis with the methodology described in Spatafora et al. (2016). Protein sequences from 49 fungi (Table 2) were downloaded either from NCBI Genomes or from JGI MycoCosm portal (Grigoriev et al. 2014). Some of the assemblies were not annotated so we performed genome annotation with GeneMark-ES v. 2.3e (Borodovsky and Lomsadze 2011) on these species: *Cystobasidiopsis lactophilus* JCM 7595, *Meira nashicola* JCM 18503, *Golubevia pallescens* JCM 5230, *Tilletia horrida* QB-1, *Pseudozyma tsukubaensis* NBRC 1940, *Sporisorium iseilematis-ciliati* BRIP 60887, *Sporisorium scitamineum* SSC39B and we validated with GAG/tbl2asn method as described above.

**Table 2:** List of genomes used for phylogenetic analysis in current study

Phylum	Name	Source of Genome Data	Reference
Basidiomycota	*Acaromyces ingoldii* MCA 4198 v1.0	JGI MycoCosm	Kijpornyongpan et al. 2018
Basidiomycota	*Agaricus bisporus var. bisporus* H97 v2.0	JGI MycoCosm	Morin et al. 2012
Basidiomycota	*Auricularia subglabra* v2.0	JGI MycoCosm	Floudas et al. 2012
Basidiomycota	*Ceraceosorus guamensis* MCA 4658 v1.0	JGI MycoCosm	Kijpornyongpan et al. 2018
Basidiomycota	*Cystobasidiopsis lactophilus* JCM 7595	NCBI (BCIO01000001.1) ^a^	Manabe et al. unpublished
Basidiomycota	*Exobasidium vaccinii* MPITM v1.0	JGI MycoCosm	Spatafora et al. unpublished
Basidiomycota	*Golubevia pallescens* JCM 5230	NCBI (BCHO01000001.1) ^a^	Manabe et al. unpublished
Basidiomycota	*Jaminaea* sp. MCA 5214 v1.0	JGI MycoCosm	Kijpornyongpan et al. 2018
Basidiomycota	*Malassezia globosa*	JGI MycoCosm	Xu et al. 2007
Basidiomycota	*Malassezia sympodialis* ATCC 42132	JGI MycoCosm	Gioti et al. 2013
Basidiomycota	*Meira miltonrushii* MCA 3882 v1.0	JGI MycoCosm	Kijpornyongpan et al. 2018
Basidiomycota	*Meira nashicola* JCM 18503	NCBI (BCJU01000001.1) ^a^	Manabe et al. unpublished
Basidiomycota	*Melanotaenium endogenum* CBS 481.91 v1.0	JGI MycoCosm	Spatafora et al. unpublished
Basidiomycota	*Mixia osmundae* IAM 14324 v1.0	JGI MycoCosm	Toome et al. 2014b
Basidiomycota	*Moesziomyces antarcticus* DSM 70725	JGI MycoCosm	Lorenz et al. 2014
Basidiomycota	*Moesziomyces aphidis* DSM 70725	JGI MycoCosm	Lorenz et al. 2014
Basidiomycota	*Pseudozyma hubeiensis* SY62	JGI MycoCosm	Konishi et al. 2013
Basidiomycota	*Pseudozyma tsukubaensis* NBRC 1940	NCBI (MAIP00000000.1) ^a^	Geiser et al. 2016
Basidiomycota	*Puccinia striiformis* f. sp. *tritici* PST-130	JGI MycoCosm	Cantu et al. 2011
Basidiomycota	*Sporisorium iseilematis-ciliati* BRIP 60887	NCBI (MJEU00000000.1) ^a^	Geiser et al. 2016
Basidiomycota	*Sporisorium reilianum* SRZ2	JGI MycoCosm	Schirawski et al. 2010
Basidiomycota	*Sporisorium scitamineum* SSC39B	NCBI (CP010913.1) ^a^	Taniguti et al. 2015
Basidiomycota	*Testicularia cyperi* MCA 3645 v1.0	JGI MycoCosm	Kijpornyongpan et al. 2018
Basidiomycota	*Tilletia caries* DAOMC 238032		current study
Basidiomycota	*Tilletia controversa* DAOMC 236426		current study
Basidiomycota	*Tilletia controversa* DAOMC 238052		current study
Basidiomycota	*Tilletia horrida* QB-1	NCBI (LAXH01000001.1) ^a^	Wang et al. 2015
Basidiomycota	*Tilletia indica* DAOMC 236408		current study
Basidiomycota	*Tilletia indica* DAOMC 236414		current study
Basidiomycota	*Tilletia indica* DAOMC 236416		current study
Basidiomycota	*Tilletia laevis* ATCC 42080		current study
Basidiomycota	*Tilletia laevis* DAOMC 238040		current study
Basidiomycota	*Tilletia walkeri* DAOMC 236422		current study
Basidiomycota	*Tilletia walkeri* DAOMC 238049		current study
Basidiomycota	*Tilletiaria anomala* UBC 951 v1.0	JGI MycoCosm	Toome et al. 2014a
Basidiomycota	*Tilletiopsis washingtonensis* MCA 4186 v1.0	JGI MycoCosm	Kijpornyongpan et al. 2018
Basidiomycota	*Tremella mesenterica* Fries v1.0	JGI MycoCosm	Floudas et al. 2012
Basidiomycota	*Ustilago hordei* Uh4857_4	JGI MycoCosm	Laurie et al. 2012
Basidiomycota	*Ustilago maydis* 521 v2.0	JGI MycoCosm	Kämper et al. 2006
Basidiomycota	*Violaceomyces palustris* SA 807 v1.0	JGI MycoCosm	Kijpornyongpan et al. 2018
Basidiomycota	*Wallemia ichthyophaga* EXF-994	JGI MycoCosm	Zajc et al. 2013
Ascomycota	*Neurospora crassa* OR74A v2.0	JGI MycoCosm	Galagan et al. 2003
Ascomycota	*Saccharomyces cerevisiae* S288C	JGI MycoCosm	Goffeau et al. 1996
Ascomycota	*Schizosaccharomyces pombe*	JGI MycoCosm	Wood et al. 2002
Blastocladiomycota	*Allomyces macrogynus* ATCC 38327 v1.0	JGI MycoCosm	Nordberg et al. 2013
Blastocladiomycota	*Blastocladiella* cf. *britannica* JEL711	JGI MycoCosm	James et al. unpublished
Blastocladiomycota	*Catenaria anguillulae* PL171 v1.0	JGI MycoCosm	Mondo et al. 2017
Chytridiomycota	*Gaertneriomyces semiglobifer* Barr 43 v1.0	JGI MycoCosm	James et al. unpublished
Chytridiomycota	*Globomyces pollinis-pini* Arg68 v1.0	JGI MycoCosm	James et al. unpublished
Chytridiomycota	*Neocallimastix californiae* G1 v1.0	JGI MycoCosm	Haitjema et al. 2017
Cryptomycota	*Rozella allomycis* CSF55 v1.0	JGI MycoCosm	James et al. 2013
Mucoromycota	*Mortierella elongata* AG-77 v2.0	JGI MycoCosm	Uehling et al. 2017
Mucoromycota	*Mucor circinelloides* CBS 277.49 v2.0	JGI MycoCosm	Corrochano et al. 2016
Mucoromycota	*Phycomyces blakesleeanus* NRRL1555 v2.0	JGI MycoCosm	Corrochano et al. 2016
Mucoromycota	*Rhizophagus irregularis* DAOM 181602 v1.0	JGI MycoCosm	Tisserant et al. 2013
Mucoromycota	*Rhizopus oryzae* v1.0	JGI MycoCosm	Ma et al. 2009
Zoopagomycota	*Basidiobolus meristosporus* CBS 931.73 v1.0	JGI MycoCosm	Mondo et al. 2017
Zoopagomycota	*Coemansia reversa* NRRL 1564 v1.0	JGI MycoCosm	Chang et al. 2015
Zoopagomycota	*Conidiobolus coronatus* NRRL 28638 v1.0	JGI MycoCosm	Chang et al. 2015

^a^ Denotes genomes that we annotated in this study because no annotations were available at the time of our analyses

Protein sequences from the input fungal species and our ten *Tilletia* genomes were searched with hmmsearch from the hmmer3.1b package (Eddy 2009) against the 192 Profile Hidden Markov Models (HMM) built from phylogenetically informative markers in Spatafora et al. (2016). Sequence alignment was performed by profile HMM using hmmalign. Poorly aligned regions were trimmed automatically with trimAl v1.4rev15 (Capella-Gutiérrez et al. 2009). A maximum likelihood phylogenetic analysis was performed using RAxML v8.2.9 (Stamatakis 2014) with the fast bootstrap method (option -f a) and 100 bootstrap replicates. The best model amino acid substitution (PROTCATAUTO) was called. All generated trees were analyzed in ASTRAL-II v4.10.10 (Mirarab and Warnow 2015) to construct a greedy consensus tree with 100 bootstrap replicates following instructions found at https://github.com/smirarab/ASTRAL. This was done to evaluate the potential conflicts among genes.

### Finding species specific genes for detection assay

To find candidate genes for species specific detection assays, orthogroups representing single copy genes that are also unique to each *Tilletia* species were identified from OrthoFinder’s output. Of note, because *T. caries* was represented by a single genome, the single copy genes unique to that species were found in the “unassigned” orthogroups in the OrthoFinder results. Using the list of gene names in the OrthoFinder output, sequences were extracted as fasta files with the filterbyname.sh script from BBTools v35 (https://jgi.doe.gov/data-and-tools/bbtools/). To verify that a given orthogroup is really unique to a given species, each fasta file was used as a query for a BLAST search against all *Tilletia* genes at both the nucleotide and amino acid level. The number of single copy and unique orthogroups found for each species is shown in Table 3. Orthogroups found to be unique at the amino acid level were considered to be better candidates. However, only one orthogroup was found at the amino acid level for *T. laevis*, so for this species, we considered those found at the nucleotide level as well. The following characteristics were considered to be more desirable in potential genes for designing primers and probes for a detection assay: complete genes (predicted with both start and stop codons) that are in the middle of a scaffold rather than at the periphery; genes that were called with at least one other gene on the same scaffold (e.g., not a singleton on a scaffold); genes that did not contain assembly gaps. These criteria were used to further narrow down the list of candidates from Table 3. Using Geneious R10 (Biomatters Ltd., Auckland, New Zealand), primers and probes were designed using very stringent parameters, with amplicon size between 100 bp to 150 bp and inside an exon, primers with a T_m_ of approximately 60 °C and roughly 23 bp long, and probes with a T_m_ of approximately 70 °C and roughly 27 bp long. The suggested primers and probes sequences for each candidate gene are reported in Table 4. These primers and probes were mapped back to all genome assemblies using the Geneious R10 mapper to verify that there was no potential cross reactivity to a species they were not designed to amplify. For each candidate gene, the protein sequence was analyzed with InterProScan (Finn et al. 2017) using the online tool at EMBL-EBI on April 14, 2018 (https://www.ebi.ac.uk/interpro/search/sequence-search), as well as blastp against the NCBI nr database on April 10, 2019, to find its putative function. Each candidate gene was analyzed with EffectorP 2.0 (Sperschneider et al. 2018), hosted on http://effectorp.csiro.au on April 10, 2019.

**Table 3:** Number of unique single copy genes of each *Tilletia* species after the all versus all BLAST verification step

		Number of single copy genes unique to a given species	
Species	Strains considered	at the nucleotide level	at the amino acid level
*Tilletia caries*	DAOMC 238032	377	72
*Tilletia controversa*	DAOMC 236426, DAOMC 238052	35	2
*Tilletia laevis*	DAOMC 238040, ATCC 42080	13	1
*Tilletia indica*	DAOMC 236416, DAOMC 236408, DAOMC 236414	61	2
*Tilletia walkeri*	DAOMC 236422, DAOMC 238049	65	5

**Table 4:** Best primers and probes sequences for targetting the best genes unique to each species to give amplicon sizes approximately between 100 bp to 150 bp

		Orthogroup putative function		Effector analysis^c^				
Species	Orthogroup ID	InterProScan^a^	NCBI blastp nr database^b^	Prediction	Probability	Forward Primer	Probe	Reverse Primer
*Tilletia caries*	OG0010723	Unknown	hypothetical protein	Non-effector	0.71	TGTCTGCTACCTTTCTTTGGGTT	CGGTCAGCATATCTAGCGTCGCAGCCT	ACCTAGTTCGCAGGAAGAATGTT
	OG0010724	Unknown	GTPase	Non-effector	0.69	TAGTGGATCGACAACGGAAAACT	GTGATGTGGCGAAAACCATCGGGAGCC	AGCTCATCAATCAGCTCGAACAG
	OG0010727	Unknown	hypothetical protein	Non-effector	0.66	CTGAAATTGCTGTCATCTGGGTG	ACCAGTCCTCGCCTACCTTGATAGCCA	AACTAGACTCTGGTTAGACGTGC
	OG0010729	Contains signal peptide	hypothetical protein	Effector	0.68	TTCTTATCTGCCCTTACCTGCTG	TATCCACGTCACTCAAGCCTTGCGCCT	CAGCCATAACAATCGCATACAGG
	OG0010739	Contains DNA binding domain	XRE family transcriptional regulator	Non-effector	0.63	CCAAAGGCGATATGAGCGTCTC	CCTGAAGCTGGTCAAGGCCGGACATCA	CTCGTCGAGGATGTCTTCAATCG
	OG0010773	Contains EamA domain	EamA family transporter	Non-effector	0.91	TCCAAATCCAGTAGCCAATGAGG	TCGAGAAGATGGCGGCGTTGTCGATCT	GGAGATTTATGTGCGGACATTGG
	OG0010825	Unknown	DUF2384 domain-containing protein	Non-effector	0.59	GCTCAAAACGATACCAGGCATAG	GCGGTTCCACCTTGCTGAGCACCTCTA	CCGCATGGAATCAGATGACATTC
	OG0010847	Unknown	hypothetical protein	Non-effector	0.81	CCTGTAGCAACTTCGTCAGATCC	GTTCGCTGCCATCGACGAGCTGCTTTG	AGCAGATCATCGTTCAGAACCTT
	OG0010878	Contains domain associated eicosanoid/glutathione metabolism	MAPEG family protein	Non-effector	0.90	CAAGATCAACCAATGGACCTTGC	GAGTGTCGGTGCTGGCAGTTTGATGGC	GGTGAGAAAGAACTCCTCATGCT
	OG0010879	Contains PDZ domain	hypothetical protein	Non-effector	0.54	TTCATCTTCAGGCTGACCAAAGT	GCGCCACAGATCGCCGGAATTGTATGA	GGAAGCGAGACGAGCAAATATTC
	OG0010908	Contains signal peptide	hypothetical protein	Effector	0.65	TGAACCATCGGCTTTCTTAGTCA	GCTCCCAACGACAGAAGCTGCTCCTCA	GATGTCACTTGCTACGGCTACTA
	OG0010935	Unknown	hypothetical protein	Unlikely effector	0.51	GACACTCGACCGCTATCTTCAAG	CCGGAAAAGGGTGCCGTCACTCAGGAA	CCGAAATCAATGCACGGTCATAT
	OG0010940	Unknown	hypothetical protein	Effector	0.66	CGTAGAGTCGCCTCGAGAAATTA	TCATGGGCCAATCCTCCAATGCGAACA	GGAGCTTATCTGTAACCGCAAAA
	OG0010947	Unknown	hypothetical protein	Unlikely effector	0.50	GATAGCCTTGATAATGCCGATGC	GAGATCCGAGACTGCCCGGCTGACGTT	TATAATCGACGATGAGCTACGGC
	OG0011035	Unknown	No significant hits found	Non-effector	0.74	GATTCAAAATCGCGGGACAGTAC	ATCAGGGCGAGCCTGTGATACACGGTG	TCCAAAGATCAAGCTGGCGATTA
	OG0011043	ABC-2 transporter	ABC transporter permease	Non-effector	0.89	CATCCATTGTTGATGCTGCTCTT	CTGACGTCGATCACCTTCAGCCTCGCT	CAGGCTCAATTGCTCGAAATTCT
	OG0011044	Aspartic peptidase	TIGR02281 family clan AA aspartic protease	Unlikely effector	0.55	CCTGAGTTCATCCGTCTGCATC	GCAGCGAACTCAGGCTCGACAAGAAGC	GATCATGCGAACCAATGTCGAG
*Tilletia controversa*	OG0009908	Unknown	hypothetical protein	Effector	0.69	CTTGACTCTCCAGTGGTCGAAG	GGAACTCCCGTCAAGACAACAGCGACG	TTCACGTACTACCTCAAGGACAG
*Tilletia laevis*	OG0009501	Unknown	hypothetical protein	Effector	0.68	CGCAAACTGGAATGGACCATACT	ATTCTACGCGCAACGGTCTGCAAGAGG	GTTGTAAACTCGATCCGCATTCG
	OG0009526	Unknown	No significant hits found	Non-effector	0.94	GTGAGGAATTACGATCTGTCCCC	TACCCGATGAGATGCGTTCGGCTGGTC	GATCCCACATACCAGAGTTCCAG
*Tilletia indica*	OG0009272	Unknown	hypothetical protein	Non-effector	0.92	GAGGACCTTCAAGATCTGACAGG	ACACCTAGGCCACTCCCTATCCAGCCA	CTGATGATCTTGCCCGGTTTTAC
*Tilletia walkeri*	OG0010415	Contains signal peptide	hypothetical protein	Unlikely effector	0.53	TCAACTACTTCGACTCCTCCTCC	CTTCCGTGATCCCGTCAACGTCGGACT	GCGACACCATCCTTAGTTGTGTA
	OG0010423	Contains signal peptide	hypothetical protein	Non-effector	0.94	ATCTTCTACCCAATCACCTGCAG	GCCCAGCAAGGTGTTTTTGGCCAAGGA	ATAGCATCACTGATGAGCGTCTG
	OG0010431	Contains signal peptide	hypothetical protein	Non-effector	0.52	TTTCGATCACAGGACCAAGGATC	ACTTGAGGCCAGGGTGTCACATAGGCG	GCACTTCTACCTTTCTACCCCTT

^a^Putative function determined with Interproscan(https://www.ebi.ac.uk/interpro/search/sequence-search)

^b^Putative function determined from NCBI’s blastp analyses on the nr database. Only results with alignment scores of > 80 were considered

^c^Effector probability determined by effectorP 2.0 (http://effectorp.csiro.au)

## RESULTS

### Genome sequencing, assembly and annotation

The raw sequencing data were assembled *de novo* into 907 to 4633 scaffolds with GC content from 54.8 to 56.7% and N50 from 11.5 Kb to 79.5 Kb. Notably, the genome assembly size ranged anywhere from smaller 24 Mb for *T. walkeri* to larger 28.1 Mb to 29.9 Mb for *T. caries*, *T. controversa, T. laevis and T. indica*. Between 92 and 98% of complete BUSCO’s were detected in all genomes suggesting that a significant portion of the total genomic information was successfully recovered. We predicted 7842 to 9952 gene models, depending on the species. The results are tabulated in Table 1.

### Genomic comparison

**Fig. 1:**
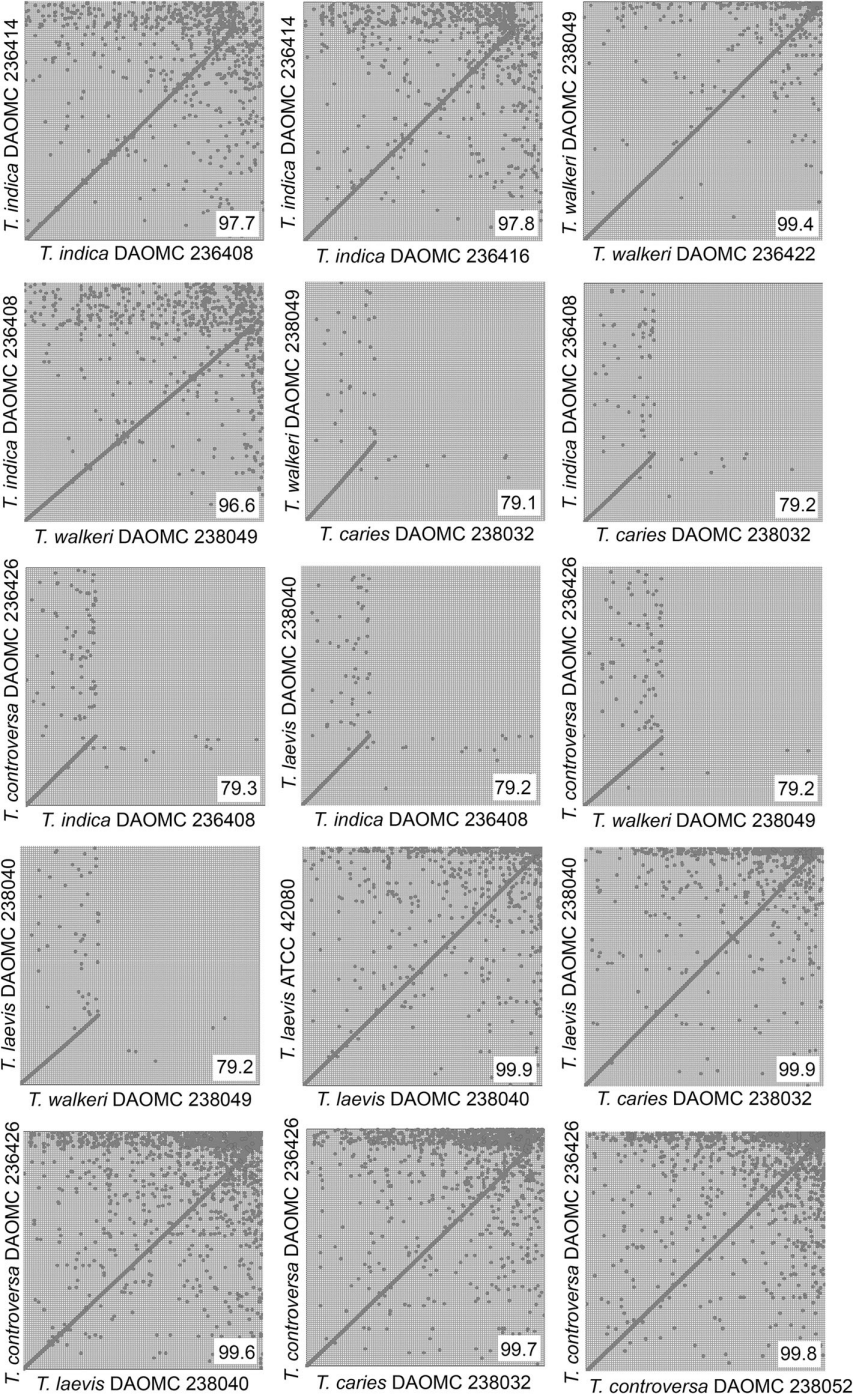
Mummerplots. Genes predicted from each genome were compared by alignment and the Average Nucleotide Identity (ANI) is shown in the bottom right corner of each comparison

Pairwise comparisons, by alignments of the genes, were performed to qualitatively assess similarities between *Tilletia* genomes and the Average Nucleotide Identity (ANI) was calculated for each pair in order to quantify similarity (Fig. 1). Gene sequences and gene content of individuals from the same species are expected to be the most similar, where almost all genes can be aligned. Species in different species complexes should share the least amount of genomic information and thus only the core *Tilletia* genes will be aligned. As expected, the mummerplots qualitatively show that *T. indica* and *T. walkeri* are well aligned with each other, as expected since they are closely related whereas *T. controversa*, *T. caries* and *T. laevis* are well aligned with each other as they reside in a separate species complex. Of note, the genome size of *T. indica* is roughly 29–30 Mb while the genome size of *T. walkeri* is only 24 Mb. In Fig. 1, the *T. indica* and *T. walkeri* gene regions are aligned but it appears that *T. indica* has extraneous sequences that *T. walkeri* does not have. This is confirmed by the fact that *T. indica* has thousands more gene models predicted than *T. walkeri* (Table 1). As for *T. controversa*, *T. caries* and *T. laevis,* they all roughly have the same genome sizes and are well aligned.

The ANI score is the average nucleotide identity shared between any two genomes (Richter and Rossello-Mora 2009) and it is an accepted metric for delimiting bacterial species. It was found that comparison of the same species should yield an ANI of > 95% (Goris et al. 2007; Richter and Rossello-Mora 2009; Jain et al. 2018). This might be a good rule of thumb to determine intra-species and inter-species boundaries. Because our mummerplots can only provide qualitative measures of similarity, we calculated the ANI score for each pair to quantify the similarities. Following the same qualitative trend as the mummerplots, the ANI score was highest when comparing strains of the same species or strains of species within a species complex (> 95%) and was lower when strains from different species complexes were compared (< 80%). Determination of ANI is not yet common practice in mycology. Recently, Lastovetsky et al. (2016) found an ANI of 92% when comparing two strains of *Rhizopus microspores* but there are not enough examples yet to find a general rule of thumb for delimiting species.

**Fig. 2:**
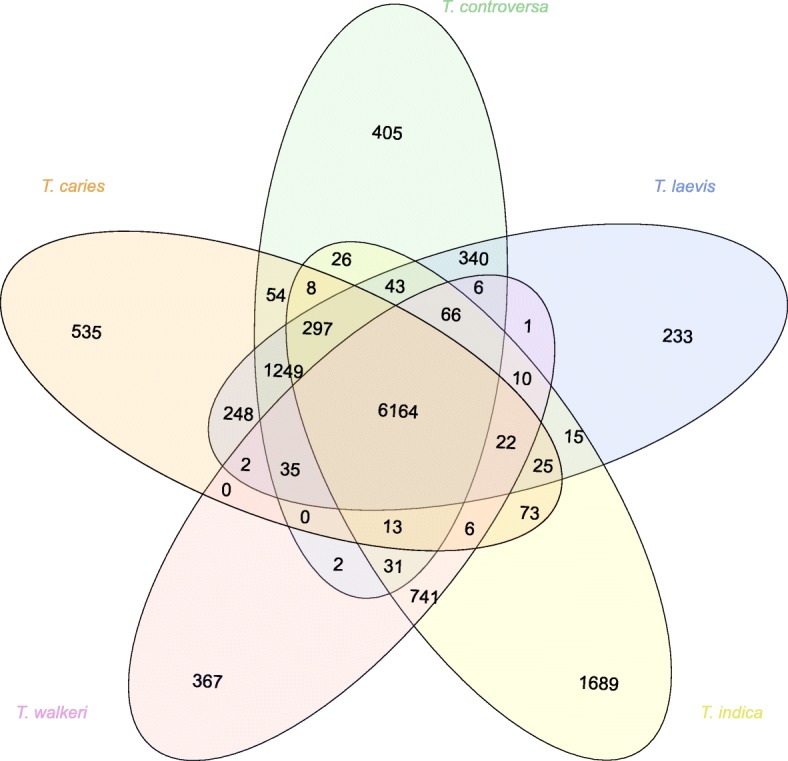
Unique and shared orthogroups between the five *Tilletia* species from OrthoFinder’s output

All protein sequences extracted from the ten *Tilletia* genomes were grouped into 10526 orthogroups. There were 6164 orthogroups that were shared among all *Tilletia* species. Of those, 4896 orthogroups were considered single copy genes, where one representative sequence was found per genome for that given orthogroup. Notably, there were 1249 orthogroups shared between *T. caries*, *T. controversa* and *T. laevis*. There were 741 orthogroups shared between *T. indica* and *T. walkeri*. The number of shared orthogroups between species is illustrated in Fig. 2 as a Venn diagram.

### Evolutionary relationships: species tree and phylogenetic placement in kingdom Fungi

**Fig. 3:**
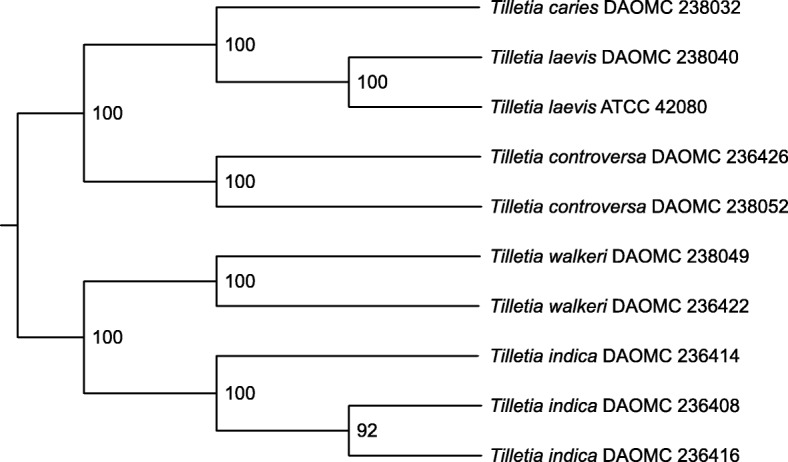
ASTRAL greedy consensus cladogram based on analyses of individual bootstrap trees of the 4896 single copy orthologous genes shared between the ten *Tilletia* strains. Support values show the percentage of bootstrap replicates that contain that branch

There is an underlying phylogenetic signal that represents the true species tree even though different parts of the genome can have different evolutionary histories leading to conflicts in gene trees. Using the sequences from the 4896 single copy genes shared among all ten *Tilletia* genomes, representing about half of the total gene models predicted, we estimated the consensus species tree using a coalescence-based method with multi-locus bootstrapping (Fig. 3) and with *Rozella allomycis* as the outgroup taxon. From these single copy loci, the total alignment length was 12.2 Mb. The total number of bases in the matrix was 120 Mb (12.2 Mb × 10 *Tilletia* genomes), where roughly 10% of those bases were gaps. For these 4896 genes, there were 2.6 million parsimony informative sites detected and the average proportion of parsimony informative sites is 0.22. This means roughly a little over one fifth of each gene contained variation useful for phylogenetic reconstruction. Based on the analysis of these 4896 genes, our concept of the *Tilletia* phylogenetic species is supported because each *Tilletia* species grouped together in a clade with 100% bootstrap support, confirming the identification using traditional Sanger sequencing (data not shown) and the results of McDonald et al. (2000) with rep-PCR DNA fingerprinting for the same DAOMC strains included in that study. It also shows the two distinct species complexes: *T. indica* and *T. walkeri* in one group, and *T. controversa*, *T. caries* and *T. laevis* in the second group.

**Fig. 4:**
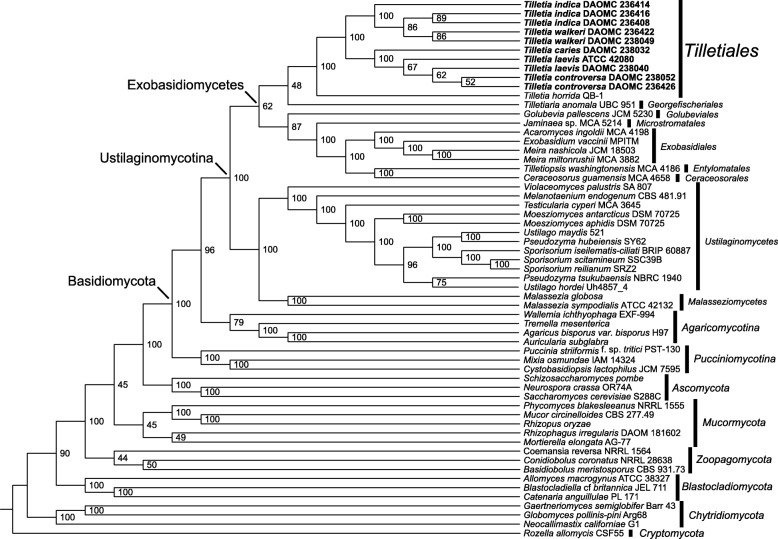
ASTRAL greedy consensus cladogram based on analyses of individual bootstrap trees for each of 192 conserved orthologous proteins placing *Tilletia* in Kingdom Fungi. Support values show the percentage of bootstrap replicates that contain that branch

We performed a phylogenomic analysis using protein sequences from 192 core conserved orthologous proteins to place *Tilletia* in the fungal kingdom (Fig. 4). This tree shows *Tilletia* in the *Ustilaginomycotina* and in the *Exobasidiomycetes* as expected. Our *Tilletia* genomes grouped with *T. horrida* in a well-supported clade representing the *Tilletiales*. The remaining taxa sampled in the class are classified in other orders. In this analysis, *T. horrida* is basal to our sequenced *Tilletia* species, which is not in conflict with the LSU analysis of Castlebury et al. (2005). These 192 genes were sufficient in separating the two *Tilletia* species complexes into two well-supported clades. They were not able to resolve some of the backbone nodes in the *Exobasidiomycetes* but groupings at the order level were consistent with the comprehensive genomics analyses of *Ustilaginomycotina* by Kijpornyongpan et al. (2018), except that theirs lacked any *Tilletiales*.

### Candidate markers for detection assay

Given the implications for trade and the export of commodities, the genes used in an assay for regulatory purposes should be precise at differentiating closely related species. The orthologous group analysis revealed some initial single copy genes that were considered unique to each species but also exist in all sequenced strains of the corresponding species: there were 535 for *T. caries*, 125 for *T. controversa*, 64 for *T. laevis*, 144 for *T. indica*, 136 for *T. walkeri*. After performing an all-versus-all BLAST search, at the nucleotide and amino acid level, as another verification step to ensure that no parts of the so-called unique genes in one species would be similar to some genes in another species, the list was narrowed down to fewer candidates (Table 3). Genes considered unique at the amino acid level were further investigated manually first. At this stage, there was only 1 such candidate for *T. laevis*. Upon further manual inspection, this candidate gene turned out to be unsuitable because it is too similar to a genomic region in *T. caries*. Thus we started looking at the 13 candidates of *T. laevis* that were found to be unique at the nucleotide level. After manual inspection and verification, only a few candidates with appropriate primer/probe sites were identified and suggested for further wet lab validation in a future study: one for *T. controversa*, two for *T. laevis*, one for *T. indica*, three for *T. walkeri* and seventeen for *T. caries* (Table 4). The designed primers and probes were mapped back to all genome assemblies, including those of *T. indica* (Sharma et al. 2016; Kumar et al. 2017; Kumar et al. 2018) and *T. horrida* (Wang et al. 2015) available on NCBI, and were found to be indeed unique for detecting the intended species for which they were designed (data not shown). To assign putative function to these candidate genes, we analyzed the protein sequence with InterProScan. Interestingly, some of the genes have totally unknown functions and some contain a signal peptide.

## DISCUSSION

The detection of quarantine *Tilletia* species on wheat is an important issue tied to trade (IPPC 2016; OEPP/EPPO 2016). In this study, we sequenced new genomes for *T. indica* and the first genomes for four other *Tilletia* species, performed phylogenomics analyses to confirm relationships, and then queried the data to identify species-specific gene regions that could be exploited for regulatory diagnostics (Table 4). Many of the DNA-based assays already published for this purpose were developed without the use of genomics data and rely on targeting commonly studied gene regions that either can easily be sequenced with existing primers or are represented in publicly available sequence databases. Those assays include real time PCR protocols probing the LSU region for *T. caries* (McNeil et al. 2004) or the ITS region for *T. indica* (Tan and Murray 2006; Tan et al. 2009; Thirumalaisamy et al. 2011; Gurjar et al. 2017). This approach uses ‘small data’ and although sometimes adequate, it might not result in extremely specific diagnostic assays. In our study, we harnessed the power of comparative genomics and ‘big data’, to perform an exhaustive search to find alternate markers that should be in theory more robust for diagnostic assays of quarantine species. Targeting genes unique to the species of interest increases the likelihood of sustainable specificity.

Currently there are six other genomes listed on GenBank for *T. indica* strains, all originating from India, none with annotations. Two are unpublished and the other four were published too recently to be included in our analyses (Sharma et al. 2016, Kumar et al. 2017, Kumar et al. 2018). There is also a genome for one *T. horrida* strain (Wang et al. 2015) originating from China. Our study adds data resources for this genus with both the genome assemblies and genome annotations of three *T. indica* strains originally from India or Pakistan, two *T. walkeri* from the USA, two *T. controversa* from Canada, one *T. caries* from the USA and two *T. laevis* from the USA or Australia. An earlier assembly of our genome for *T. indica* DAOMC 236416, made available in 2016, has already been used to help improve the *T. indica* draft genome assembly of Kumar et al. (2018).

Rather than focus on analyses of effectors and CAZymes, as has been done by others (Kumar et al. 2017; Kijpornyongpan et al. 2018), we chose to focus our efforts on finding a comparative genomics approach to analyze the data contained in the ten *Tilletia* genomes and to find unique genes for the design of detection assays, which is something that previously could not be investigated for *Tilletia*. The draft assembly of *T indica* strain TiK_1, reported by Kumar et al. (2017), contained 10957 contigs and an improved assembly of the same strain was published later with 787 scaffolds (Kumar et al. 2018), whereas our *T. indica* genomes were assembled into 2555 to 4624 scaffolds. Kumar et al. (2017) reported about 11500 gene models annotated without transcriptome data. In the improved assembly of strain TiK_1, those authors also obtained 9209 protein coding gene models, but it was unclear whether transcriptome data was considered in the annotation procedure. We obtained roughly 9500 gene models using transcriptome data. The *T. indica* TiK_1 strain was not included in our analyses because the annotations were not available for download and we already had data for three strains of *T. indica* collected in different years or different countries*,* which were adequate to achieve our objective. To verify that this did not result in excluding a strain of *T. indica* that was genetically divergent to our strains, we performed a core genome alignment and phylogeny, using Parsnp (Treangen et al. 2014), and found that *T. indica* TiK_1 groups together in one phylogenetic clade with all of our *T. indica* strains (Nguyen unpublished data), thus confirming that adding TiK_1 would not likely have further improved our results.

The genomes of *T. walkeri* from our study are an important contribution for future in-depth genomic studies focused on *T. walkeri* and *T. indica*. Although their ITS sequences are almost identical, their genomes are markedly different in size, at approximately 30 Mb for *Tilletia indica* and about 24 Mb for *T. walkeri*. Our genome annotation indicates that *T. indica* has thousands of extra genes compared to its sister species (Table 1, Fig. 1), and the importance of these genes could be investigated further, eg. for potential roles in pathogenicity. The study by Kijpornyongpan et al. (2018) included analyses of gene conservation among members of the *Ustilaginomycotina* and found that the pathogenicity genes previously identified for *Ustilago maydis* were limited in distribution among the genomes of other taxa. Their analyses lacked representatives from the *Tilletiales*, but the authors hypothesized that fungi in this order would also lack these genes, and may have developed alternative mechanisms. We performed a preliminary analysis on our genome annotations (Nguyen unpublished data) employing the same detection methodology, and found that the *Tilletia* species sequenced from this study lack the known smut pathogenicity genes, except for the Srt1-high affinity sucrose transporter (gene UM02374 in *U. maydis*). This would be another potential avenue for investigation in future genetic studies.

The five species clustered into well-supported clades in the phylogenetic analysis with 4896 single copy orthologous genes (Fig. 3), suggesting that our concept of the phylogenetic species in these two complexes held together with multi-locus genome data. After performing manual verification, we proposed primers and probes for a reduced list of species-specific genes (Table 4). We only sequenced one strain of *T. caries* so these 17 suggested candidate markers may not actually exist in all strains of *T. caries* in nature and could be unique to DAOMC 238032. Each of the other species had two or more sequenced genomes so the suggested genes should be more robust. We performed an InterProScan analysis and a blastp analysis against the NCBI nr database to attempt to predict the function of these genes. Many of them had unknown function while few contained a signal peptide at the N terminus. Some were predicted by EffectorP 2.0 to be effectors with probabilities between 0.65 and 0.69. Generally, families of candidate effectors were identified on the basis of predicted N-terminal signal peptides, their small size, and lack of similarity to other proteins (Petre and Kamoun 2014). However, these predictions are strictly in silico and functional wet lab experiments would need to be carried out to validate real function.

One prerequisite for the development of robust, sensitive and specific assays is a comprehensive understanding of the systematic and biological relationships of the targets and their closest relatives. Species concepts should be clearly defined and the strains used in the design of any diagnostic assay accurately identified. This aspect remains a challenge for the DB and CD pathogens given their propensity for hybridization with each other and with some other *Tilletia* species. Conspecificity of the DB pathogen and the two CB species was proposed by Russell and Mills (1994), based on results of previous and new genetic, biochemical, physiologic and morphological data, and discussed by others (Shi et al. 1996; Bao 2010). Inter-species hybridization among the DB and CB species was postulated to explain observed morphological intermediates (Wilcoxson and Saari 1996) but also demonstrated in the laboratory in planta or axenic culture (Trail and Mills 1990), between those species and *T. fusca* (Carris and Gray 1994) and between DB and *T. bromi* (Pimentel et al. 2000). Comprehensive population genetics studies, which have so far been limited to RAPD and RFLP methods, also provided evidence of natural hybridization among the 95 isolates tested, representing DB, CB and *T. fusca* var. *bromi-tectorum* (Shi et al. 1996). Despite these findings, which underscore the challenges for accurate diagnostics, DB and CB continue to be considered distinct, in large part because of differences in disease etiology and perceived risk (Wilcoxson and Saari 1996). Treating them as distinct phylogenetic species is supported by our phylogenomics analysis (Fig. 3) where there is strong bootstrap support for CB as one clade and DB as a separate clade.

A second prerequisite is acquiring a sufficient number of representatives of varied provenance so that assay validation is tested against a broad sampling of genotypic variation. Progress is an iterative process as samples and data resources are compiled and analyzed. The next step is to gather sufficient numbers of field samples of the wheat-associated *Tilletia* species and their close relatives for validation testing of these markers and other markers not selected by our methods for screening the genes recovered in our orthologous groups analysis. Adding redundancy by identifying a set of markers or gene regions for development of multiple assays at more than one level of phylogenetic resolution would increase confidence in diagnostic assessments by providing checks against false negatives and for previously unknown or undetected genomic variation. Comparative genomics is the most promising approach for accomplishing this goal. In addition, it was envisioned that the genomic resources generated in this study would contribute in the future to other assay development efforts using SNP (single nucleotide polymorphism) discovery and also to population genetics studies of the DB/CB species complex.

## CONCLUSION

We sequenced and annotated ten genomes of five *Tilletia* species in two separate species complexes: three strains of *T. indica* and two strains of *T. walkeri* in one complex, and two strains of *T. controversa*, one strain of *T. caries* and two strains of *T. laevis* in another complex. Through comparative genomic approaches, we identified gene candidates, and designed primers and probes that are potentially suitable for differentiating each species and are to be validated in future wet-lab studies.

## Additional file

Additional file 1: **Table S1.** NGS datasets generated in the study. (XLSX 12 kb)

## Data Availability

Sequencing data are uploaded to NCBI and accession numbers listed in Additional file 1: Table S1. Cultures listed in Table 1 are deposited in the Canadian Collection of Fungal Cultures (DAOMC) or the American Type Culture Collection (ATCC).

## Abbreviations

ANI: Average Nucleotide Identity
CB: Common Bunt
DB: Dwarf Bunt
